# Supplementary material for: AAMP and MTSS1 Are Novel Negative Regulators of Endothelial Barrier Function Identified in a Proteomics Screen
Source: Cells. 2024 Sep 25;13(19):1609. doi: 10.3390/cells13191609 (PMC11476176; doi:10.3390/cells13191609)
Supplement: Supplementary file 1 [file cells-13-01609-s001.zip › cells-3140236-supplementary.pdf]

Supplementary Materials

AAMP and MTSS1 are novel negative regulators of endothelial barrier function identified in a proteomics screen

Fabienne Podieh[1], Max C. Overboom[1], Jaco C. Knol[2], Sander R. Piersma[2], Richard Goeij-de Haas[2], Thang V. Pham[2], Connie R. Jimenez[2], Peter L. Hordijk[1]*

[1]*Department of Physiology, Amsterdam Cardiovascular Science, Microcirculation, Amsterdam UMC, Amsterdam, The Netherlands*

[2]*Department of Medical Oncology, OncoProteomics Laboratory, Cancer Center Amsterdam, Amsterdam UMC, Amsterdam, The Netherlands*

[*]*Corresponding author: p.hordijk@amsterdamumc.nl*

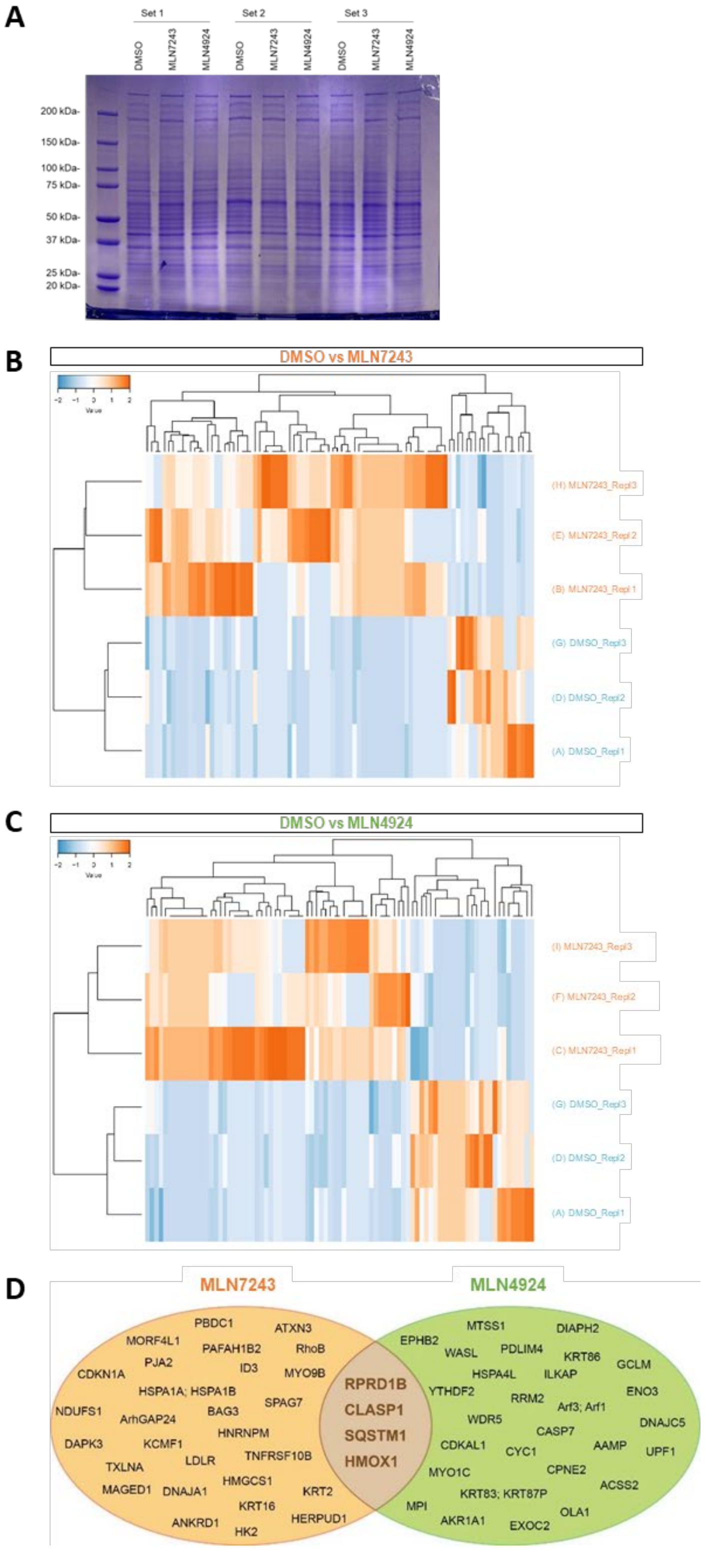

Figure S1 DMSO- and inhibitor-treated samples show differential protein expression clusters (**A**) For sample preparation for proteomics, HUVECs were treated with DMSO, 500 nM MLN7243 or 500 nM MLN4924 in three independent experiments. Protein lysates were separated by SDS-PAGE and stained with Coomassie. (**B, C**) Heat maps show supervised clustering of proteomics samples: three lysates of DMSO-treated ECs and (**B**) three lysates of MLN7243-treated ECs or (**C**) three lysates of MLN4924-treated ECs. (**D**) VENN diagram with

32 proteins after analysis of DMSO vs MLN7243 (figure 1A) and 32 proteins after analysis of DMSO vs MLN4924 (figure 1B).

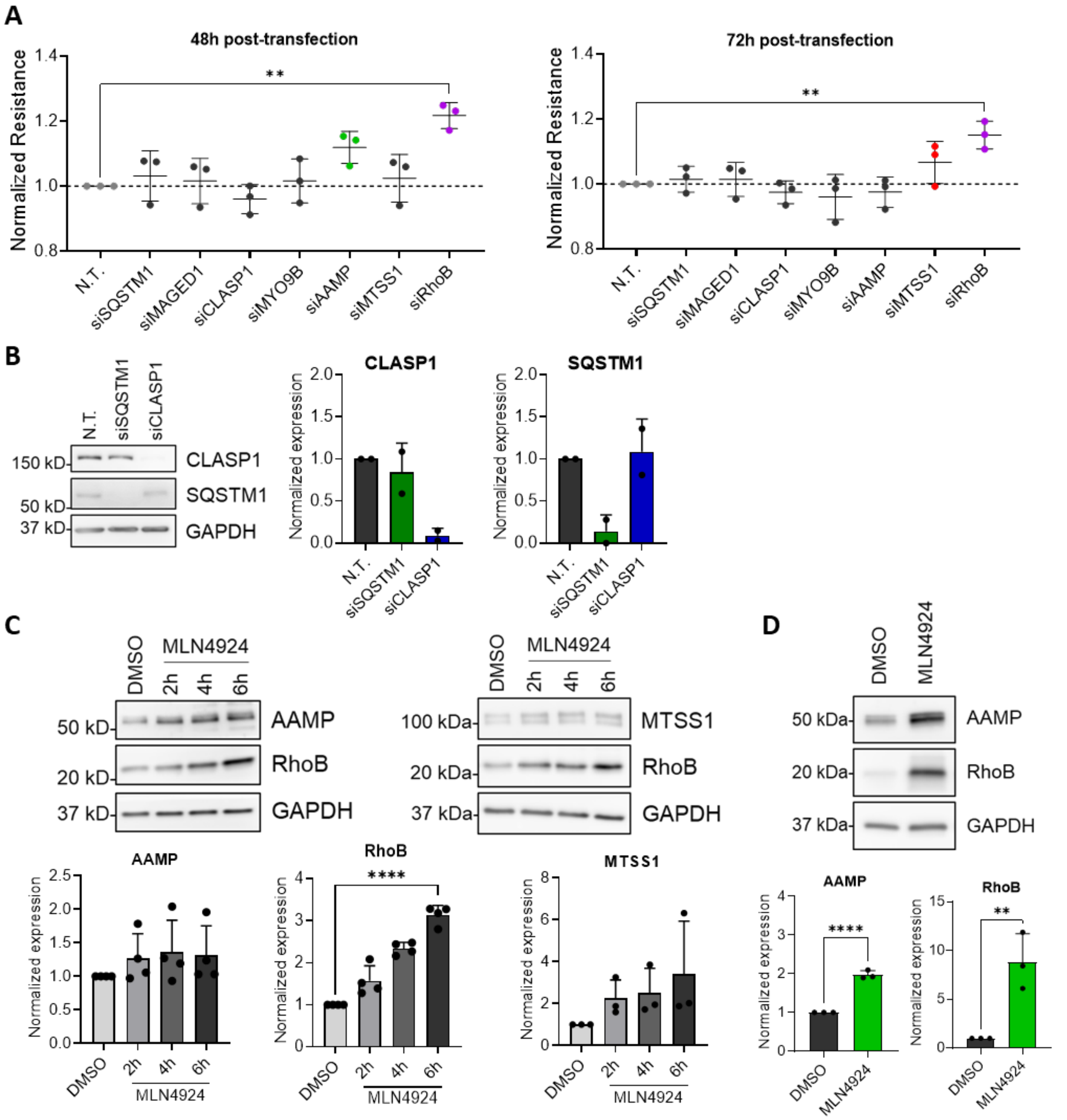

Figure S2 Blocking Cullin E3 ligases leads to accumulation of AAMP and MTSS1

(**A**) HUVECs were transfected with N.T., siSQSTM1, siMAGED1, siCLASP1, siMYO9B, siAAMP, siMTSS1 or siRhoB. Resistance of HUVECs 48 h and 72 h post-transfection is shown. Data are presented as mean ± SD and normalized to respective N.T., n = 3. Graphs represent the normalized data from figure 2A. (**B**) HUVECs were transfected with N.T., siSQSTM1 or siCLASP1. Western blot analysis of CLASP1 and SQSTM1 expression. Bar graphs show quantified CLASP1 and SQSTM1 expression normalized to respective GAPDH and N.T. Data are presented as mean + SD, n = 2. (**C**) Western blot analysis of AAMP, MTSS1 and RhoB expression after 500 nM MLN4924 for indicated time points. Bar graphs show

quantification of AAMP, MTSS1 and RhoB expression normalized to respective GAPDH and DMSO. Data are presented as mean + SD, n=3-4. (**D**) Western blot analysis of AAMP and RhoB expression of proteomics samples, hence after 500 nM MLN4924 for 6h. Bar graphs show quantification of AAMP and RhoB expression normalized to respective GAPDH and DMSO. Data are presented as mean + SD, n=3. ** p < 0,01; **** p < 0,0001; HUVEC, human umbilical vein endothelial cell; N.T., non-targeting siRNA

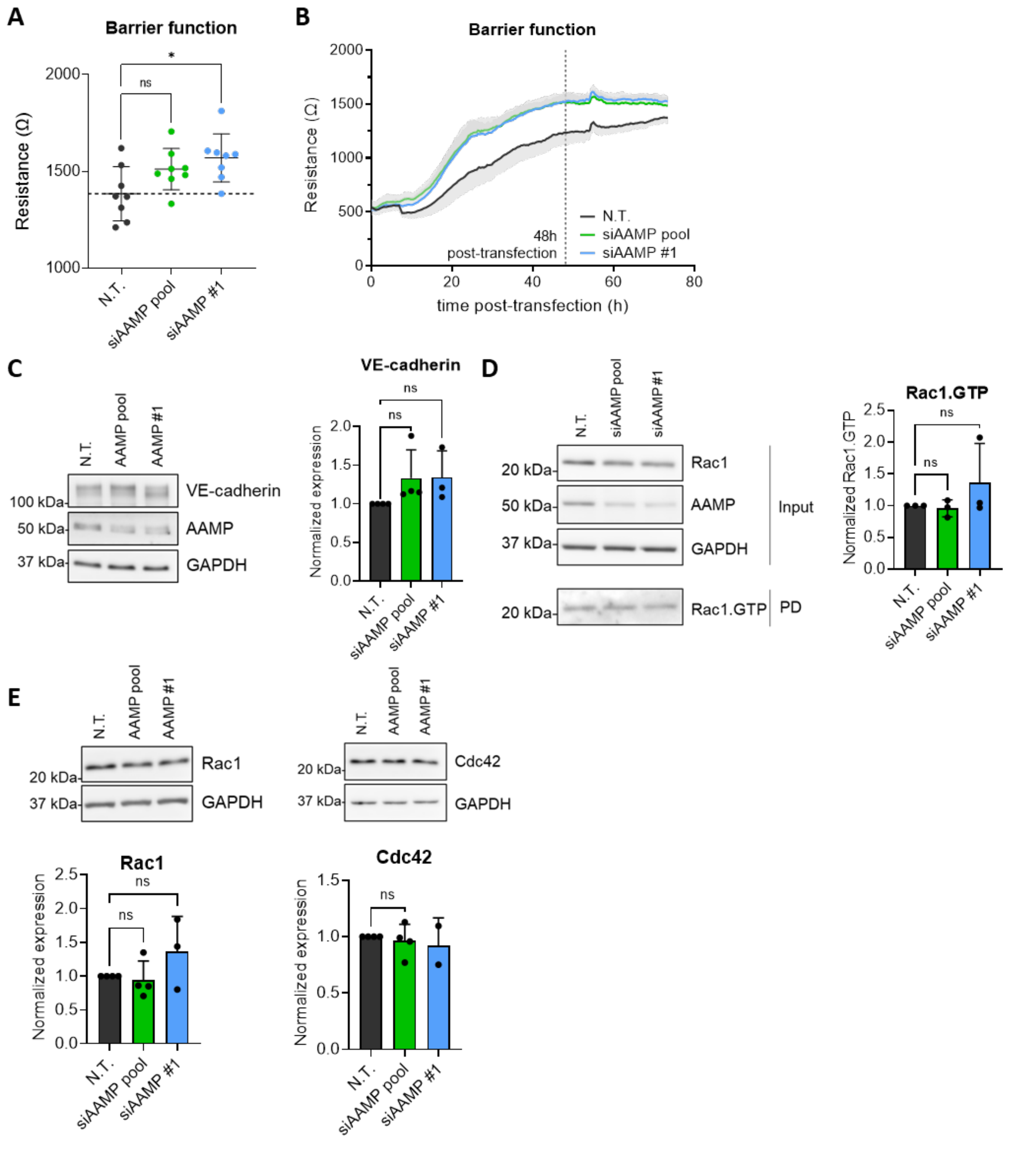

Figure S3: VE-cadherin, Rac1 and Cdc42 remained mostly unaffected by depletion of AAMP

(**A**-**E**) HUVECs were transfected with N.T., siAAMP pool or siAAMP #1. (**A**) Resistance of HUVECs 48 h post-transfection. Absolute values from figure 3A. Data are presented as mean ± SD, n = 8. (**B**) Resistance of HUVECs over time. The graph represents the time course of one representative experiment depicted in A, as mean + SD. (**C**) Western blot analysis for VE-cadherin expression. Bar graph shows quantification of VE-cadherin expression normalized to GAPDH and N.T. Data are presented as mean + SD, n = 2-3. (**D**) CRIB pulldown followed by Western blot analysis for the GTP-bound, active form of Rac1. Bar graph shows quantification of Rac1.GTP normalized to Rac1 and GAPDH. Data are presented as mean + SD, n = 3. (**E**) Western blot analysis for Rac1 and Cdc42 expression. Bar graphs show quantification of Rac1 and Cdc42 expression normalized to GAPDH and N.T. Data are presented as mean + SD, n = 1-3. * p < 0,05; HUVEC, human umbilical vein endothelial cell; N.T., non-targeting siRNA; CRIB, Cdc42/Rac Interactive Binding, PD, pulldown; ns, non-significant

---

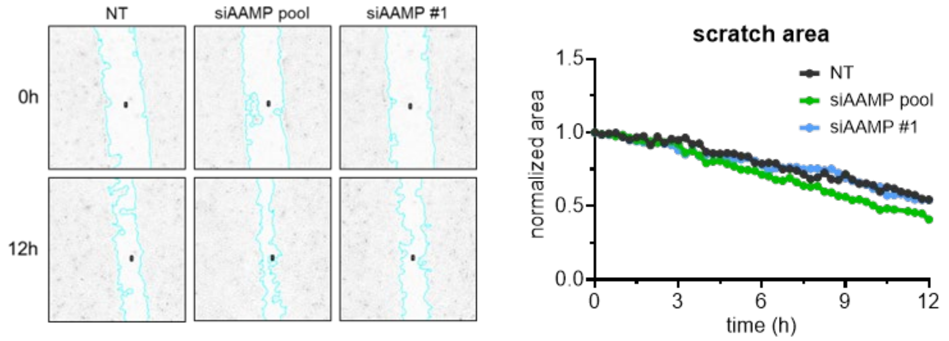

Figure S4: Scratch assay with AAMP-depleted HUVECs
After transfection of HUVEC with N.T., siAAMP pool or siAAMP #1, the monolayer was manually wounded. The area of the scratch was monitored for 12 h. HUVEC, human umbilical vein endothelial cell; N.T., non-targeting siRNA

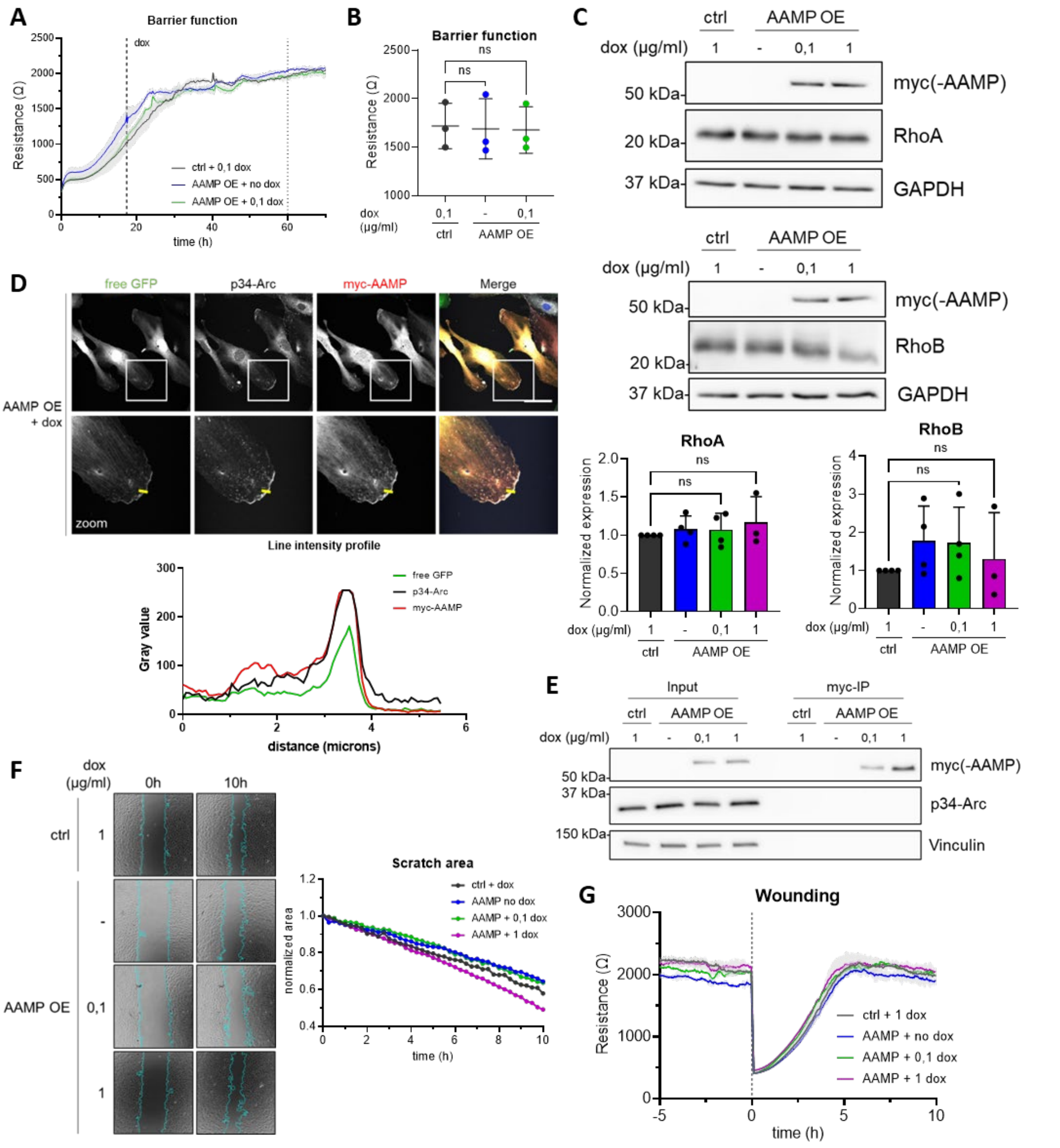

Figure S5: AAMP overexpression does not affect endothelial barrier function, total RhoA/B levels or EC migration

(**A-B**) HUVECs were transduced with ctrl or AAMP lentivirus. (**A**) Resistance was measured over time and HUVECs treated with indicated concentration of doxycycline. (**B**) Scatter blot shows absolute resistance values of stable endothelial barrier (60 h). Data are presented as mean ± SD, n = 3. (**C-G**) HUVECs were transduced with ctrl or AAMP lentivirus and treated with indicated concentrations of doxycycline for 24 h. (**C**) Western blot analysis for myc-AAMP, RhoA and RhoB expression. Bar graph shows quantification of RhoA and RhoB expression normalized to GAPDH and ctrl + 1 µg/ml dox. Data are presented as mean + SD, n = 3-4. (**D**) Immunofluorescent staining of sub-confluent HUVECs for GFP (green), p34-Arc (white), myc-

AAMP (red) and counterstained with DAPI (blue). Scale bar represents 50 µm. Graph shows gray values of all channels along the yellow line drawn in zoom images. (**E**) Myc-co-IP followed by Western blot analysis for myc-AAMP and p34-Arc. (**F**) HUVEC monolayer was manually scratched and imaged for 12 h. (**G**) Resistance was measured while HUVEC monolayer was wounded by a high electrical current. HUVEC, human umbilical vein endothelial cell; ctrl, control; dox, doxycycline; OE, overexpression; ns, non-significant
